# Melatonin Levels in Serum and Ascitic Fluid of Patients with Hepatic Encephalopathy

**DOI:** 10.1155/2012/510764

**Published:** 2012-12-30

**Authors:** Cezary Chojnacki, Marek Romanowski, Katarzyna Winczyk, Janusz Błasiak, Jan Chojnacki

**Affiliations:** ^1^Department of Gastroenterology, Medical University of Lodz, 1 Haller's Square, 90-647 Lodz, Poland; ^2^Department of Neuroendocrinology, Medical University of Lodz, 1/3 Sterlinga street, 91-425 Lodz, Poland; ^3^Department of Molecular Genetics, University of Lodz, 141/143 Pomorska street, 90-237 Lodz, Poland

## Abstract

Cirrhotic patients exhibit disturbed melatonin homeostasis, which may lead to sleep disturbances, but an influence on the hepatic encephalopathy has not been elucidated. *Aim*. In the present study, the association of melatonin levels in serum and ascitic fluid and ammonia concentration related to the intensity of hepatic encephalopathy (HE) was investigated. *Material and Methods*. The study included 90 alcoholic patients with hepatic encephalopathy and 30 healthy volunteers (C). Patients were divided in three groups according to 0–4 West-Haven Score: HE_1_ (*n* = 28), HE_2_ (*n* = 30), and HE_3_ (*n* = 32). Melatonin was measured by radioimmune assay. *Results*. In fasting patients with hepatic encephalopathy we noted higher melatonin serum levels [pg/mL] than in healthy subjects groups: C—11.3 ± 3.9, HE_1_ – 34.3 ± 12.2 (*P* < 0.01), HE_2_—54.8 ± 23.9, and HE_3_—119.8 ± 96.4 (*P* < 0.001). No correlation between melatonin and ammonia levels was found. Melatonin was detected in ascetic fluid in 24 patients of group HE_2_ and 27 patients of group HE_2_ of hepatic encephalopathy. *Conclusions*. Our results suggest that high blood levels of melatonin in cirrhotic liver patients may account for some of the clinical manifestations of hepatic encephalopathy, for example, daytime sleepiness, fatigue.

## 1. Introduction

Hepatic encephalopathy (HE) is a complex neuropsychiatric syndrome which is characterized by disturbances in behavioral and consciousness as well as neurological symptoms. Detailed pathophysiology of this disease remains unknown, but some of the pathogenetic factors have been identified. This includes disturbances of hepatocyte function because of toxic substances produced during metabolism. As result, toxic substances are transported via the blood from the portal vein into various organs including brain where they induce metabolic disturbances. 

Ammonia has been identified as a factor of hepatic encephalopathy pathogenesis. It disturbs enzymatic processes in brain tissue, inhibits the activity of acetylcholine and dopamine, and increases accumulation of false neurotransmitters [1]. However, high levels of ammonia were not observed in all hepatic encephalopathy patients, indicating that other chemical substances should be also considered as pathogenetic factors. Possible candidates are the excess of methionine and its mercaptan derivatives as well as aromatic amino acids (phenylalanine, tyrosine, tryptophan, and methionine). These agents block the synthesis of physiological neurotransmitters and induce the production of false ones, including octopamine, which may replace normal neurotransmitters, especially noradrenaline and dopamine in synapses. This causes muscle tremors and psychoemotional disturbances [2, 3]. Disturbances in consciousness may also be a consequence of increased concentration of some neurotransmitters, including gamma-aminobutyric acid (GABA) and other biologically active compounds. Melatonin has beneficial effects on a variety of central nervous system (CNS) diseases [4, 5]. However, the influence of melatonin on CNS in individuals with liver insufficiency has not been sufficiently recognized. 

 Melatonin is released by the pineal gland according to the circadian rhythm, and its peak levels are near the middle of the dark phase [6]. Nocturnal concentrations of melatonin are severalfold higher than in the daytime [7]. Due to its high lipophilicity melatonin released from pinealocytes easily penetrates all cells and enters the body fluids [8, 9]. The gastrointestinal tract (GIT) is also a source of melatonin, from where it is released following various stimuli, including alimentary signals. In this location it seems to be synthesized when L-tryptophan is supplied in the diet [10, 11]. The precise mechanisms of synthesis and regulation of melatonin in the GIT are not known. Melatonin released from enteroendocrine cells (ECs) plays an important enteroprotective role via paracrine mechanisms [10–12]. Melatonin is metabolized in enterocytes by cytochrome CYP1B1 fraction [13]. The majority of melatonin is transported to the liver through hepatic portal vein [14, 15]. About 90% of alimentary tract-released melatonin is inactivated during the first passage through the liver [16]. As in the got, melatonin is metabolized in hepatocytes by the cytochrome P-450 enzymes (CYP1A1 and CYP1A2) to 6-sulfatoxymelatonin and 6-hydroxymelatonin glucuronide, which are removed with the urine [17–19]. A fraction of melatonin may be released into bile in its unchanged form, since the concentration of indoleamine in the bile is extremely high [20, 21]. The melatonin receptors are expressed in human gallbladder epithelia [22]. High concentrations of melatonin in the bile may prevent the epithelium of bile tracts and intestines from injury by biliary acids.

Melatonin release and metabolism seem to be affected by various pathological states, especially those associated with liver disease. Disturbances in circadian rhythm of melatonin release from the pineal gland with its peak in the morning were observed in patients with cirrhosis [23, 24]. It was suggested that this occurred as a result of hepatic insufficiency-related metabolic disturbances [25]. Similar changes were observed in rats after installation of portosystemic shunt [26, 27]. In this situation, administration of neomycin correct at the rhythm to a melatonin release [28]. These findings promoted conclusion that increased concentrations of ammonia in the blood exerted toxic effects on brain structures, including the pineal gland, and changed the rhythm of melatonin release [29–31]. This may indicate that the increase of melatonin concentration in the morning may depend on the ability of liver to metabolize it; this is possible in patients with hepatic insufficiency. A fivefold increase in the concentration of melatonin was observed in the fasting state in cirrhotic patients with portal hypertension as compared with controls. The concentration of melatonin increased almost four-fold after test meal when taken with 10 mg melatonin [32]. Such high concentration of melatonin in hepatic insufficiency might result from its disturbed metabolism and its discharge from portal to systemic circulation. 

In the present study we evaluated the melatonin concentration in the blood and ascitic fluid in patients with hepatic encephalopathy.

## 2. Material and Methods

### 2.1. Patients

#### 2.1.1. Including Criteria

Ninety liver cirrhosis patients with postal hypertension and ascites (grade B, C acc. to Child-Pugh Score) [33] and thirty sex- and age-matched healthy volunteers were enrolled in this study. The study was approved by the Local Ethics Committee (RNN272/05/KB), and each patient gave written consent.  Table 1 presents general characteristics of the subjects.

The diagnosis of liver cirrhosis was based on the results of social, clinical, imaging (USG, panendoscopy, and CT), and laboratory investigations. Liver biopsy with histopathological analysis was performed in 11 patients. Each patient abused alcohol for 6–21 years. All patients also underwent neurological, psychological, psychiatric and in 58 patients psychometrical examinations, including the number connection tests NCT-A and NCT-B and the line tracing test (LTT) in order to determine the intensity of hepatic encephalopathy [34]. The results of these examinations allowed for categorization of patients into group HE_1_ (*n* = 28), HE_2_ (*n* = 30), and HE_3_ (*n* = 32) according to the West-Haven criteria [35].

#### 2.1.2. Excluding Criteria

Viral hepatitis, hepatic coma, delirium, psychic illness, renal insufficiency, and post-surgical state.

### 2.2. Analytical Procedures

The following biochemical examinations were performed on the patients: blood cell count, bilirubin, urea, creatinine, ALT, AST, GGTP, ALP, glucose, cholesterol, INR, prothrombin, APTT, albumins, globulins, GFR, HBsAg, and anti-HCV. In particular, ammonia in serum was performed by enzymatic method using glutaminic dehydrogenase.

The concentration of albumins and globulins as well as the number and kind of cells was determined in ascitic fluid. Sixteen patients were additionally examined to exclude bacterial infection. Blood was taken from basilic vein, and ascitic fluid was obtained by abdominocentesis and collected at the same time, at 09:00 h at which time the patients were in fasting state and in the daylight for 2 hrs. Three days before the examination, the patients were on the same standard diet consisting of 3 × 400 mL (1800 kcal) Nutridrink (Nutricia, Poland) (1800 kcal) and 1500 mL mineral water. Samples of serum and ascitic fluid were centrifuged and kept at −70°C until the measurement of melatonin; samples were never kept longer than 4 months before the assays were performed. The concentration of melatonin was determined with the Melatonin Direct RIA (IBL, Hamburg, Germany) kit with the detection limit 2.5 pg/mL.

### 2.3. Statistical Analysis

To compare serum melatonin levels among all groups the nonparametric Mann-Whitney test was applied, since the distribution of the analyzed parameters significantly differed from the normal distribution. To compare melatonin levels between groups with 1–3 degree of hepatic encephalopathy the nonparametric Kruskal-Wallis test was used with the subsequent Mann-Whitney test. The Mann-Whitney test was also used to compare melatonin concentrations in blood and ascitic fluid. The correlation between the concentrations of ammonia and melatonin in blood was evaluated by linear regression equation with the Pearson's correlation coefficient (*r*). All statistical analyses were performed with the use of STATISTICA, v. 9.0 package (Tulsa, OK, USA).

## 3. Results

**Figure 1:**
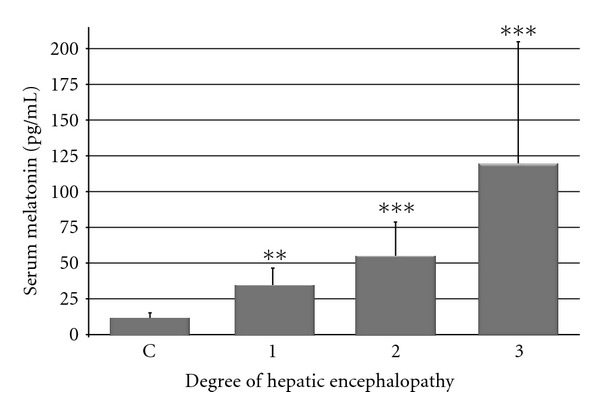
Serum melatonin levels in healthy subject (C) and in cirrhotic patients with different (1, 2, 3) degree of hepatic encephalopathy—according to West-Haven Score. **P < 0.01, ***P < 0.001.

Serum melatonin level in healthy subjects was 11.3 ± 3.9 pg/mL, and it was significantly higher in cirrhotic patients and strongly depended on the degree of hepatic insufficiency (Figure 1). In patients classified into group HE_1_ the level of melatonin was 34.3 ± 12.2 pg/mL (*P* < 0.01), in group HE_2_—54.8 ± 23.9 pg/mL (*P* < 0.001), and in group HE_3_—119.8 ± 96.4 pg/mL (*P* < 0.001). 

Serum ammonia concentrations (*μ*g/dL) were in groups: C—30.4 ± 8.9, HE_1_—52.6 ± 26.0 (*P* < 0.05), HE_2_—74.2 ± 30.6 (*P* < 0.001), and HE_3_—109.5 ± 38.9 (*P* < 0.001). 

**Figure 2:**
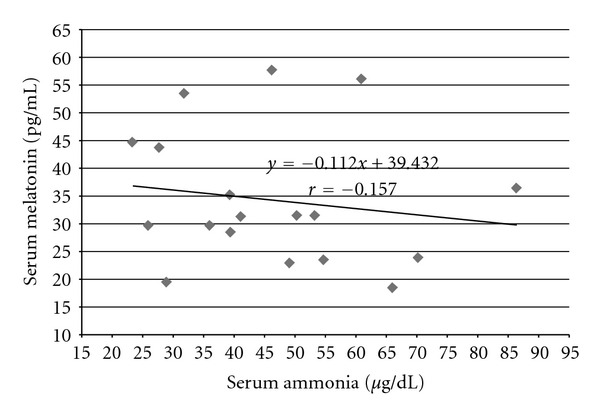
Correlation between ammonia and melatonin serum levels in cirrhotic patients with 1st degree of hepatic encephalopathy (group HE_1_) according to West-Haven Score; *y*: regression equation, *r*: correlation coefficient, statistically insignificant.

**Figure 3:**
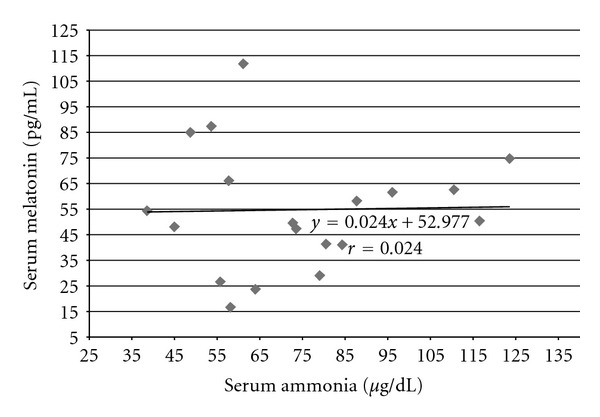
Correlation between ammonia and melatonin serum levels in cirrhotic patients with 2nd degree of hepatic encephalopathy (group HE_2_) according to West-Haven Score; *y*: regression equation, *r*: correlation coefficient, statistically insignificant.

**Figure 4:**
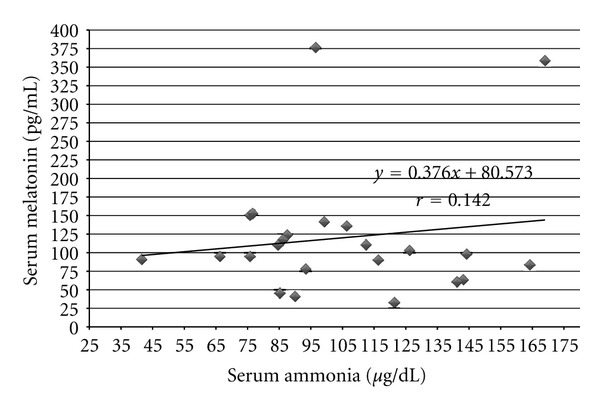
Correlation between ammonia and melatonin serum levels in cirrhotic patients with 3rd degree of hepatic encephalopathy (group HE_3_) according to West-Haven Score; *y*: regression equation, *r*: correlation coefficient, statistically insignificant.

We did not find any correlation between melatonin and ammonia levels in all groups of patients with hepatic encephalopathy. Value of correlation coefficient (*r*) in group HE_1_ was *r* = −0.157 (Figure 2), in group HE_2_ (*r* = 0.024) (Figure 3), and in group HE_3_  (*r* = 0.142) (Figure 4).

**Figure 5:**
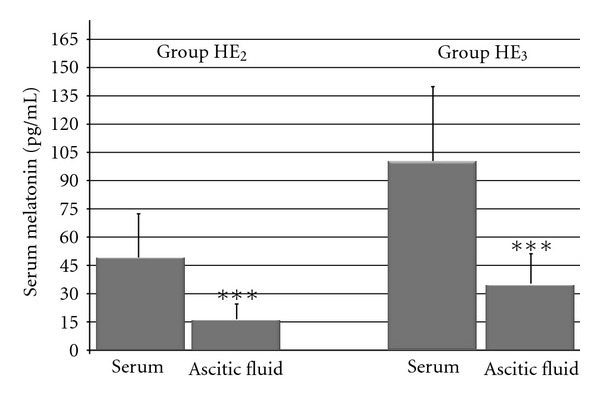
Comparison of melatonin concentration in serum and ascetic fluid in cirrhotic patients with 2nd (group HE_2_, *n* = 24) and 3rd (group HE_3_, *n* = 25) degrees of hepatic encephalopathy. ***P < 0.001. There are significant differences between mean values obtained in ascitic fluid in groups HE_2_ and HE_3_—*P* < 0.01.

Increased melatonin concentration in ascitic fluid was detected in 24 patients out of 30 (80%) in group HE_2_ and 27 patients out of 32 (84,3%) in group HE_3_, and it was 16.4 ± 14.9 pg/mL and 45.5 ± 48.6 pg/mL, respectively (*P* < 0.05, Figure 5). Melatonin concentrations in both groups were about threefold lower than in serum (*P* < 0.001, Figure 5).

Melatonin levels in ascitic fluid of two patients of group HE_3_ exceeded 600 pg/mL (609 and 632 pg/mL) and were similar to their respective serum concentrations (641 and 667 pg/mL); those were excluded from the statistical analyses. The remaining 11 patients displayed no melatonin in ascitic fluid.

## 4. Discussion

Results of many studies suggest that melatonin, both produced in the pineal gland and GIT, reaches the liver via blood circulation. Its concentration in biliary fluid is manyfold higher than diurnal concentrations in blood [20, 21]. Melatonin fulfills many important functions before its degradation, especially in reference to antioxidative defense. Mitochondria of hepatocytes are sites of intensive metabolism which lead to detoxification of many substances which are harmful for the organism. However, these detoxification processes are associated with the release of ROS, which can damage hepatocytes. Melatonin protects these cells against detrimental action of ROS because of its multiple antioxidative actions [36–38]. Melatonin protects hepatocytes against derivatives of oxygen metabolism and improves mitochondrial functions [39]. Likewise, melatonin displays preventive properties against harmful consequences of age-related changes in oxygen metabolism in rats [40]. The hepatoprotective action of melatonin was also documented against ionizing radiation [41], carbon tetrachloride [42], cold storage and reperfusion [43–46], hepatotoxic compounds [47–51], and bile duct ligation [52–54]. Melatonin also exerts anti-inflammatory actions in bile ducts and the pancreas [55, 56]. These beneficial actions of melatonin are attributed to its antioxidant properties [57, 58] although its receptor-mediated effects may also be beneficial [59]. Melatonin also decreases the production of proinflammatory cytokines, IL-1*β* and TNF-*α* and inhibits fibrogenesis in the liver [60, 61]. These results suggest the possibility for testing melatonin for therapeutic purposes. Melatonin protected against fat-rich diet-induced liver cirrhosis in rats [62]. A decrease in the activity of gamma-glutamyl transpeptidase and the level of proinflammatory cytokines was observed in patients with nonalcoholic steatohepatitis (NASH) after 4 weeks of L-tryptophan (the precursor of melatonin) administration [63]. A progressive reduction in the level of aminotransferases ALT and AST has been observed in patients with NASH after daily (2 × 5 mg) melatonin administration [64]. Melatonin at a dose of 50 mg/kg body weight improved the postoperational state of patients after partial liver resection [65]. 

It would be expected that the highest concentration of melatonin in cirrhotic patients would occur in the morning following the night time rise. The elevated serum levels are likely due to the decrease in the metabolic clearance rate, probably related to reduced liver blood flow, lowered activity of 6 *β*-hydroxylase, and competition with bilirubin in the intrahepatic transport system [66]. Results obtained in various laboratories are ambiguous, however, which may result from the differences in the patient-recruiting procedures. It has been observed that morning melatonin concentrations are higher in patients with minimal hepatic insufficiency compared to patients with a higher degree of this disease [67]. The highest concentration of morning melatonin, 102 pg/mL, was observed in the study in which only patients classified into group B according to Child-Pugh criteria were enrolled. We obtained a substantially lower concentration of morning melatonin, that is, 48.7 pg/mL, in the current report performed in a similar population of patients with hepatic insufficiency (group B), but this value was significantly higher in patients with more serious hepatic insufficiency. The difference may be related to the differences between the classifications, since our group C patients displayed extreme hepatic insufficiency. Moreover, we did not observe any correlation between the results of laboratory tests and concentrations of melatonin in blood; in particular no association between the concentrations of melatonin and ammonia was seen. This may result from different degrees of disturbances in the metabolic pathway of urea and melatonin. We did observe a relationship between the degree of liver encephalopathy and melatonin concentrations; however that high daytime melatonin concentration could result in daytime sleepiness and fatigue [68–70]. The involvement of melatonin in the pathogenesis of liver encephalopathy should be considered in patients with a high melatonin concentrations both in the day and at night. 

Interestingly, the great majority of patients with portal hypertension had measurable levels of melatonin in their ascitic fluid. The mechanism of its leakage of melatonin from portal veins into peritoneal cavity is not completely clear. Also it is unknown why the concentration of melatonin in ascitic fluid in some cirrhotic patients was very high, while this indoleamine was not detected in this fluid in other patients. It is worth noting that in every case, melatonin concentration in blood was higher than in ascitic fluid. Melatonin in the ascitic fluid may be derived from the blood, from the GIT, or from both. Certainly, in patients with hepatic insufficiency the leakage of melatonin from these two sites could occur. A better understanding of the transfer of GIT and/or blood melatonin into ascitic fluid of patients with severe liver damage should be examined in greater depth.

In summary, our results indicate that increased concentration of melatonin in blood of patients with liver cirrhosis may be the consequence of both hepatic insufficiency and transport of melatonin from gastrointestinal tract to systemic circulation through the portosystemic shunts. High concentrations of melatonin in blood may influence the clinical features of hepatic encephalopathy. 

## Figures and Tables

**Table 1:** Characteristics of the subjects enrolled in the study (mean values ± SEM).

Feature/parameters	Cirrhotic patients	Healthy subjects
Age (years)	44.1 ± 9.9	43.1 ± 6.8
Sex (F, M)	F-24, M-66	F-11, M-19
Bilirubin (mg/dL)	8.3 ± 7.1	0.7 ± 0.2
Ammonia (*μ*g/dL)	90.2 ± 41.0	30.4 ± 8.9
Albumins (g/dL)	3.1 ± 1.4	5.4 ± 0.6
AST (U/L)	81.6 ± 78.0	21.0 ± 4.8
ALT (U/L)	102.3 ± 121.6	23.1 ± 5.9
GGTP (U/L)	135.6 ± 90.4	26.0 ± 6.1
ALP (U/L)	49.4 ± 30.2	37.4 ± 10.8
GFR (mL/min)	97.0 ± 11.9	108.9 ± 9.6

AST: aspartate aminotransferase; ALT: alanine aminotransferase; GGTP: gamma-glutamyl transpeptidase; ALP: alkaline phosphatase; GFR: glomerular filtration rate.

## References

[1] Butterworth RF (1996). The neurobiology of hepatic encephalopathy. *Seminars in Liver Disease*.

[2] Weissenborn K, Ennen JC, Schomerus H, Rückert N, Hecker H (2001). Neuropsychological characterization of hepatic encephalopathy. *Journal of Hepatology*.

[3] Ortiz M, Córdoba J, Jacas C, Flavià M, Esteban R, Guardia J (2006). Neuropsychological abnormalities in cirrhosis include learning impairment. *Journal of Hepatology*.

[4] Xiong YF, Chen Q, Chen J, Zhou J, Wang HX (2011). Melatonin reduces the impairment of axonal transport and axonopathy induced by calyculin A. *Journal of Pineal Research*.

[5] Singhal NK, Srivastava G, Patel DK, Jain SK, Singh MP (2011). Melatonin or silymarin reduces maneb- and paraquat-induced Parkinsons disease phenotype in the mouse. *Journal of Pineal Research*.

[6] Stehle JH, Saade A, Rawashdeh O (2011). A survey of molecular details in the human pineal gland in the light of phylogeny, structure, function and chronobiological diseases. *Journal of Pineal Research*.

[7] Reiter RJ, Tan DX (2003). What constitutes a physiological concentration of melatonin?. *Journal of Pineal Research*.

[8] Venegas C, García JA, Escames G (2012). Extrapineal melatonin: analysis of its subcellular distribution and daily fluctuations. *Journal of Pineal Research*.

[9] Tan DX, Manchester LC, Sanchez-Barcelo E, Mediavilla MD, Reiter RJ (2010). Significance of high levels of endogenous melatonin in mammalian cerebrospinal fluid and in the central nervous system. *Current Neuropharmacology*.

[10] Raikhlin NT, Kvetnoy IM (1976). Melatonin and enterochromaffin cells. *Acta Histochemica*.

[11] Huether G, Poeggeler B, Reimer A, George A (1992). Effect of tryptophan administration on circulating melatonin levels in chicks and rats: evidence for stimulation of melatonin synthesis and release in the gastrointestinal tract. *Life Sciences*.

[12] Bubenik GA (2002). Gastrointestinal melatonin: localization, function, and clinical relevance. *Digestive Diseases and Sciences*.

[13] Gibson P, Gill JH, Khan PA (2003). Cytochrome P450 1B1 (CYP1B1) is overexpressed in human colon adenocarcinomas relative to normal colon: implications for drug development. *Molecular Cancer Therapeutics*.

[14] Kvetnoy IM, Ingel IE, Kvetnaia TV (2002). Gastrointestinal melatonin: cellular identification and biological role. *Neuroendocrinology Letters*.

[15] Reiter RJ, Tan DX, Mayo JC, Sainz RM, Leon J, Bandyopadhyay D (2003). Neurally-mediated and neurally-independent beneficial actions of melatonin in the gastrointestinal tract. *Journal of Physiology and Pharmacology*.

[16] Bubenik GA, Pang SF, Cockshut JR (2000). Circadian variation of portal, arterial and venous blood levels of melatonin in pigs and its relationship to food intake and sleep. *Journal of Pineal Research*.

[17] Lane EA, Noss HB (1985). Pharmacokinetics of melatonin in man: first pass hepatic metabolism. *Journal of Clinical Endocrinology and Metabolism*.

[18] Facciolá G, Hidestrand M, von Bahr C, Tybring G (2001). Cytochrome P450 isoforms involved in melatonin metabolism in human liver microsomes. *European Journal of Clinical Pharmacology*.

[19] Ma X, Idle JR, Krausz KW, Gonzalez FJ (2005). Metabolism of melatonin by human cytochromes P450. *Drug Metabolism and Disposition*.

[20] Tan DX, Manchester LC, Reiter RJ, Qi W, Hanes MA, Farley NJ (1999). High physiological levels of melatonin in the bile of mammals. *Life Sciences*.

[21] Messner M, Huether G, Lorf T, Ramadori G, Schwörer H (2001). Presence of melatonin in the human hepatobiliary-gastrointestinal tract. *Life Sciences*.

[22] Aust S, Thalhammer T, Humpeler S (2004). The melatonin receptor subtype MT1 is expressed in human gallbladder epithelia. *Journal of Pineal Research*.

[23] Ardizzi A, Grugni G, Saglietti G, Morabito F (1998). Circadian rhythm of melatonin (aMT) in liver cirrhosis. *Minerva Medica*.

[24] Montagnese S, Middleton B, Mani AR, Skene DJ, Morgan MY (2010). On the origin and the consequences of circadian abnormalities in patients with cirrhosis. *American Journal of Gastroenterology*.

[25] Steindl PE, Ferenci P, Marktl W (1997). Impaired hepatic catabolism of melatonin in cirrhosis. *Annals of Internal Medicine*.

[26] Zee PC, Mehta R, Turek FW, Blei AT (1991). Portacaval anastomosis disrupts circadian locomotor activity and pineal melatonin rhythms in rats. *Brain Research*.

[27] Coy DL, Mehta R, Zee P, Salchli F, Turek FW, Blei AT (1992). Portal-systemic shunting and the disruption of circadian locomotor activity in the rat. *Gastroenterology*.

[28] Finn B, Shah V, Gottstein J (1993). Neomycin improves a disrupted circadian rhythm in rats after portacaval anastomosis. *Hepatogastroenterolgy*.

[29] Ducis J (1994). Effect of ammonia and R05-4864 on melatonin release in pineal. *Journal of Neurochemistry*.

[30] Steindl PE, Finn B, Bendok B, Rothke S, Zee PC, Blei AT (1995). Disruption of the diurnal rhythm of plasma melatonin in cirrhosis. *Annals of Internal Medicine*.

[31] Lewy AJ, Ahmed S, Latham Jackson JM, Sack RL (1992). Melatonin shifts human circadian rhythms according to a phase-response curve. *Chronobiology International*.

[32] Celinski K, Konturek PC, Slomka M (2009). Altered basal and postprandial plasma melatonin, gastrin, ghrelin, leptin and insulin in patients with liver cirrhosis and portal hypertension without and with oral administration of melatonin or tryptophan. *Journal of Pineal Research*.

[33] Suman A, Barnes DS, Zein NN, Levinthal GN, Connor JT, Carey WD (2004). Predicting outcome after cardiac surgery in patients with cirrhosis: a comparison of Child-Pugh and MELD scores. *Clinical Gastroenterology and Hepatology*.

[34] Montagnese S, Amodio P, Morgan MY (2004). Methods for diagnosing hepatic encephalopathy in patients with cirrhosis: a multidimensional approach. *Metabolic Brain Disease*.

[35] Ferenci P, Lockwood A, Mullen K, Tarter R, Weissenborn K, Blei AT (2002). Hepatic encephalopathy—definition, nomenclature, diagnosis, and quantification: Final report of the Working Party at the 11th World Congresses of Gastroenterology, Vienna, 1998. *Hepatology*.

[36] Tan DX, Chen LD, Poeggeler B (1993). Melatonin: a potent endogenous hydroxyl radical scavenger. *Endocrine Journal*.

[37] Rodriguez C, Mayo JC, Sainz RM (2004). Regulation of antioxidant enzymes: a significant role for melatonin. *Journal of Pineal Research*.

[38] Galano A, Tan DX, Reiter RJ (2011). Melatonin as a natural ally against oxidative stress: a physicochemical examination. *Journal of Pineal Research*.

[39] Letelier ME, Jara-Sandoval J, Molina-Berríos A, Faúndez M, Aracena-Parks P, Aguilera F (2010). Melatonin protects the cytochrome P450 system through a novel antioxidant mechanism. *Chemico-Biological Interactions*.

[40] Castillo C, Salazar V, Ariznavarreta C, Vara E, Tresguerres JAF (2005). Effect of melatonin administration on parameters related to oxidative damage in hepatocytes isolated from old Wistar rats. *Journal of Pineal Research*.

[41] Koc M, Taysi S, Buyukokuroglu ME, Bakan N (2003). Melatonin protects rat liver against irradiation-induced oxidative injury. *Journal of Radiation Research*.

[42] Daniels WM, Reiter RJ, Melchiorri D, Sewerynek E, Pablos MI, Ortiz GG (1995). Melatonin counteracts lipid peroxidation induced by carbon tetrachloride but does not restore glucose-6 phosphatase activity. *Journal of Pineal Research*.

[43] Sewerynek E, Reiter RJ, Melchiorri D, Ortiz GG, Lewinski A (1996). Oxidative damage in the liver induced by ischemia-reperfusion: protection by melatonin. *Hepato-Gastroenterology*.

[44] Bülbüller N, Cetinkaya Z, Akkus MA (2003). The effects of melatonin and prostaglandin E1 analogue on experimental hepatic ischaemia reperfusion damage. *International Journal of Clinical Practice*.

[45] Vairetti M, Ferrigno A, Bertone R (2005). Exogenous melatonin enhances bile flow and ATP levels after cold storage and reperfusion in rat liver: implications for liver transplantation. *Journal of Pineal Research*.

[46] Zaoualí MA, Reiter RJ, Padrissa-Altés S (2011). Melatonin protects steatotic and nonsteatotic liver grafts against cold ischemia and reperfusion injury. *Journal of Pineal Research*.

[47] Tahan V, Ozaras R, Canbakan B (2004). Melatonin reduces dimethylnitrosamine-induced liver fibrosis in rats. *Journal of Pineal Research*.

[48] Túnez I, Muñoz MC, Villavicencio MA (2005). Hepato- and neurotoxicity induced by thioacetamide: protective effects of melatonin and dimethylsulfoxide. *Pharmacological Research*.

[49] Cruz A, Padillo FJ, Torres E (2005). Melatonin prevents experimental liver cirrhosis induced by thioacetamide in rats. *Journal of Pineal Research*.

[50] Wang H, Wei W, Wang NP (2005). Melatonin ameliorates carbon tetrachloride-induced hepatic fibrogenesis in rats via inhibition of oxidative stress. *Life Sciences*.

[51] Chen X, Zhang C, Zhao M (2011). Melatonin alleviates lipopolysaccharide-induced hepatic SREBP-1c activation and lipid accumulation in mice. *Journal of Pineal Research*.

[52] Ohta Y, Kongo M, Kishikawa T (2003). Melatonin exerts a therapeutic effect on cholestatic liver injury in rats with bile duct ligation. *Journal of Pineal Research*.

[53] Padillo FJ, Cruz A, Navarrete C (2004). Melatonin prevents oxidative stress and hepatocyte cell death induced by experimental cholestasis. *Free Radical Research*.

[54] Esrefoglu M, Gül M, Emre MH, Polat A, Selimoglu MA (2005). Protective effect of low dose of melatonin against cholestatic oxidative stress after common bile duct ligation in rats. *World Journal of Gastroenterology*.

[55] Barlas A, Çevik H, Arbak S (2004). Melatonin protects against pancreaticobiliary inflammation and associated remote organ injury in rats: role of neutrophils. *Journal of Pineal Research*.

[56] Wang H, Wei W, Shen YX (2004). Protective effect of melatonin against liver injury in mice induced by Bacillus Calmette-Guerin plus lipopolysaccharide. *World Journal of Gastroenterology*.

[57] Reiter RJ, Paredes SD, Manchester LC, Tan DX (2009). Reducing oxidative/nitrosative stress: a newly-discovered genre for melatonin. *Critical Reviews in Biochemistry and Molecular Biology*.

[58] Sahna E, Parlakpinar H, Vardi N, Ciğremis Y, Acet A (2004). Efficacy of melatonin as protectant against oxidative stress and structural changes in liver tissue in pinealectomized rats. *Acta Histochemica*.

[59] Mathes AM (2010). Hepatoprotective actions of melatonin: possible mediation by melatonin receptors. *World Journal of Gastroenterology*.

[60] Hong RT, Xu JM, Mei Q (2009). Melatonin ameliorates experimental hepatic fibrosis induced by carbon tetrachloride in rats. *World Journal of Gastroenterology*.

[61] Tahan G, Akin H, Aydogan F (2010). Melatonin ameliorates liver fibrosis induced by bile-duct ligation in rats. *Canadian Journal of Surgery*.

[62] Pan M, Song YL, Xu JM, Gan HZ (2006). Melatonin ameliorates nonalcoholic fatty liver induced by high-fat diet in rats. *Journal of Pineal Research*.

[63] Cichoz-Lach H, Celinski K, Konturek PC (2010). The effects of L-tryptophan and melatonin on selected biochemical parameters in patients with steatohepatitis. *Journal of Physiology and Pharmacology*.

[64] Gonciarz M, Gonciarz Z, Bielanski W (2010). The pilot study of 3-month course of melatonin treatment of patients with nonalcoholic steatohepatitis: effect on plasma levels of liver enzymes, lipids and melatonin. *Journal of Physiology and Pharmacology*.

[65] Nickkholgh A, Schneider H, Sobirey M (2011). The use of high-dose melatonin in liver resection is safe: first clinical experience. *Journal of Pineal Research*.

[66] Iguchi H, Kato KI, Ibayashi H (1982). Melatonin serum levels and metabolic clearance rate in patients with liver cirrhosis. *Journal of Clinical Endocrinology and Metabolism*.

[67] Velissaris D, Karamouzos V, Polychronopoulos P, Karanikolas M (2009). Chronotypology and melatonin alterations in minimal hepatic encephalopathy. *Journal of Circadian Rhythms*.

[68] Steindl PE, Finn B, Bendok B (1997). Changes in the 24-hour rhythm of plasma melatonin in patients with liver cirrhosis—relation to sleep architecture. *Wien Klin Wochenschr*.

[69] Córdoba J, Cabrera J, Lataif L, Penev P, Zee P, Blei AT (1998). High prevalence of sleep disturbance in cirrhosis. *Hepatology*.

[70] Montagnese S, Middleton B, Mani AR, Skene DJ, Morgan MY (2009). Sleep and circadian abnormalities in patients with cirrhosis: features of delayed sleep phase syndrome?. *Metabolic Brain Disease*.

